# A new compound in the BEDT-TTF family [BEDT-TTF = bis­(ethyl­enedi­thio)­tetra­thia­fulvalene] with a tetra­thio­cyanato­cuprate(II) anion, (BEDT-TTF)_4_[Cu(NCS)_4_]

**DOI:** 10.1107/S2056989018015293

**Published:** 2018-11-09

**Authors:** Christophe Faulmann, Benoît Cormary, Lydie Valade, Kane Jacob, Dominique de Caro

**Affiliations:** a CNRS, LCC (Laboratoire de Chimie de Coordination), 205 route de Narbonne, BP44099, 31077 Toulouse Cedex 4, France, Université de Toulouse, UPS, INPT, 31077 Toulouse Cedex 4, France

**Keywords:** crystal structure, BEDT-TTF, Cu(NCS)_2_, superconductor, pseudo-κ arrangement

## Abstract

A new phase combining BEDT-TTF and [Cu(NCS)_4_]^2–^ as the counter-anion, namely bis­[bis(ethyl­enedi­thio)­tetra­thia­fulvalenium] tetra­thio­cyanato­cuprate(II) bis­[bis(ethyl­ene­di­thio)­tetra­thia­fulvalene], (C_10_H_8_S_8_)_2_[Cu(NCS)_4_]·2C_10_H_8_S_8_ or (BEDT-TTF)_4_[Cu(NCS)_4_] was obtained during a galvanostatic electrocrystallization process. As previously observed with BEDT-TTF-based compounds with oxalatometallate anions, the BEDT-TTF mol­ecules in (BEDT-TTF)_4_[Cu(NCS)_4_] exhibit the so-called pseudo-κ arrangement, with two BEDT-TTF mol­ecules being positively charged and two electronically neutral. The bond lengths and angles in the two unique BEDT-TTF mol­ecules differ slightly. The crystal structure consists of layers of BEDT-TTF mol­ecules extending parallel to (001). The width of this layer corresponds to the length of the *a* axis [16.9036 (17) Å]. The BEDT-TTF layers are separated by layers of centrosymmetric square-planar [Cu(NCS)_4_]^2–^ dianions.

## Abstract 2

The new compound (BEDT-TTF)_4_[Cu(NCS)_4_] based on the organic donor BEDT-TTF [bis­(ethyl­enedi­thio)­tetra­thia­fulvalene] has been obtained during a galvanostatic electrocrystallization process with Cu(NCS). It exhibits a pseudo-κ arrangement, never observed up to now in the BEDT-TTF family with thio­cyanato­cuprate(II) anions.

## Chemical context   

**Figure 1:**
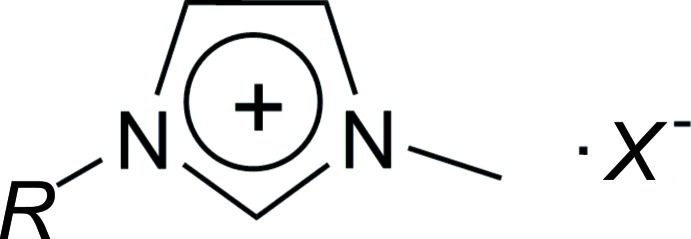
Examples of ionic liquids with the imidazolium fragment. For example, *R* = but­yl: BMI*M* ^+^; *R* = eth­yl: EMI*M* ^+^; *X* = PF_6_^−^, BF_4_^−^, SCN^−^.

For several years, we have been inter­ested in synthesizing mol­ecular (super)conductors as nanoparticles (Chtioui-Gay *et al.*, 2016 ▸; Valade *et al.*, 2016 ▸; de Caro, Jacob *et al.*, 2013 ▸; de Caro, Souque *et al.*, 2013 ▸; de Caro *et al.*, 2014 ▸; Winter *et al.*, 2015 ▸) in order to study the effects of size reduction on the properties of this kind of material. As there are numerous structuring agents such as ionic liquids based, for instance, on imidazolium cations (Fig. 1 ▸), and long alkyl chains in ammonium salts or neutral amines, it is possible to obtain nanoparticles of these materials, either by electrochemical oxidation or by chemical reaction. Recently, we have focused on BEDT-TTF-based compounds [BEDT-TTF is bis­(ethyl­enedi­thio)­tetra­thia­fulvalene]. The BEDT-TTF family is one of the most studied in the field of mol­ecular superconductors because it exhibits the largest number of superconductors with *T*_c_ above 10 K (Ishiguro *et al.*, 1998 ▸). During the planned electrosynthesis of (BEDT-TTF)_2_[Cu(NCS)_2_] as nanoparticles from BEDT-TTF and Cu(SCN) in the presence of (EMIM)(SCN) (EMIM = 1-ethyl-3-methyl­imidazolium), a few crystals were formed as a minor product besides the desired powder as the main phase. A structure determination of these crystals revealed a new salt-like compound, based on the BEDT-TTF donor and the [Cu(NCS)_4_]^2–^ dianion, namely pseudo-*κ*-(BEDT-TTF)_4_[Cu(NCS)_4_].
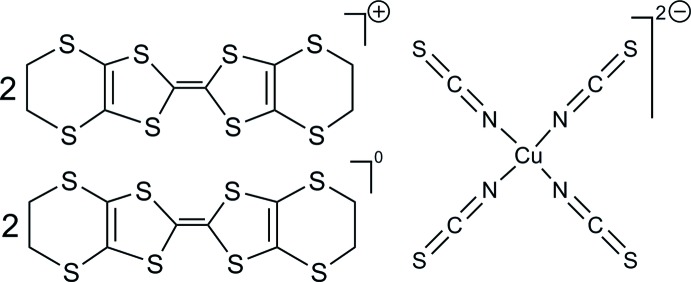


## Structural commentary   

**Figure 2:**
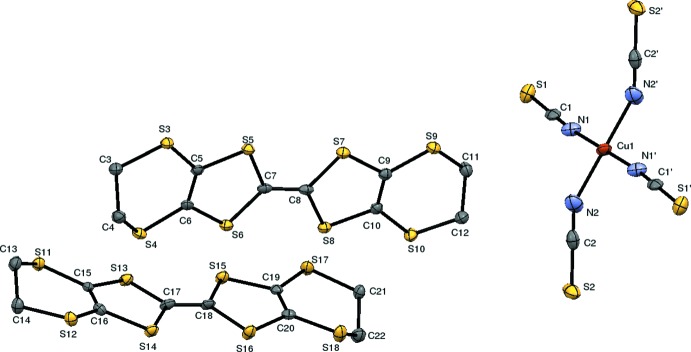
Molecular structure of (BEDT-TTF)_4_[Cu(NCS)_4_], showing the BEDT-TTF mol­ecule involved in the formation of a dimer (top), and the other BEDT-TTF mol­ecule (bottom), as well as the centrosymmetric [Cu(NCS)_4_]^2–^ dianion. Displacement ellipsoids are drawn at the 50% probability level. H atoms have been omitted for clarity. Primed atoms are generated by the symmetry operation (−*x*, 1 − *y*, 2 − *z*).

**Figure 3:**
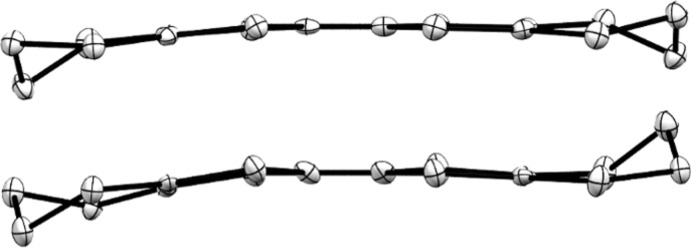
Side view of the BEDT-TTF mol­ecules (top: in the dimer; bottom: the other mol­ecule). Displacement ellipsoids are drawn at the 50% probability level.

The asymmetric unit of the title salt contains two well-ordered BEDT-TTF mol­ecules and one Cu(NCS)_2_ entity with the Cu^II^ cation lying on an inversion centre (Fig. 2 ▸). This results in the composition (BEDT-TTF)_4_[Cu(NCS)_4_], and thus is different from the well-known *κ*-phase (BEDT-TTF)_2_[Cu(NCS)_2_] (Hiramatsu *et al.*, 2015 ▸; Schultz *et al.* 1991 ▸; Urayama *et al.*, 1988 ▸) and also from (BEDT-TTF)[Cu_2_(NCS)_3_] (Geiser *et al.*, 1988 ▸). One of the two BEDT-TTF mol­ecules (central bond C7—C8) forms a dimer that is related through an inversion centre, whereas the other BEDT-TTF mol­ecules (central bond C17—C18) are farther away from each other. To our knowledge, this feature has not been observed within the (BEDT-TTF)[Cu(NCS)_*x*_] family, but it has been found in BEDT-TTF compounds with tris-(oxalato)metallate anions, such as (BEDT-TTF)_4_[*AM*(C_2_O_4_)_3_]·solv. (*A* = K, NH_4_, H_3_O; *M* = Fe, Cr, Co, Ru; solv. = benzo­nitrile, 1,2-di­chloro­benzene, bromo­benzene) (Kurmoo *et al.*, 1995 ▸; Martin *et al.*, 2001 ▸; Prokhorova *et al.*, 2011 ▸, 2013 ▸). The latter compounds are representatives of the pseudo *κ*-phase where the two independent BEDT-TTF mol­ecules show some slight structural differences. Similarly, the bond lengths within the central C_2_S_4_ core in the BEDT-TTF mol­ecules of the title salt deviate by up to 0.035 Å. The bond lengths in the TTF core are indicative of the degree of charge in this family of BEDT-TTF compounds. According to Guionneau *et al.* (1997 ▸), this allows the charge *Q* of the two BEDT-TTF mol­ecules in the title salt to be calculated. Whereas each BEDT-TTF mol­ecule in the dimer carries a charge of +1 (*Q* = 0.83), the other BEDT-TTF mol­ecule is neutral (*Q* = 0.18). Not only do the bond lengths of the BEDT-TTF mol­ecules in the title salt show some differences, but the overall shape of the mol­ecules also differs. The BEDT-TTF mol­ecule in the dimer deviates less from planarity [r.m.s. deviation of 0.0853 Å neglecting the outer ethyl­ene bridges, with the largest deviation being 0.1579 (19) Å for S5] than the other BEDT-TTF mol­ecule [r.m.s. deviation of 0.1431 Å; highest deviation = 0.3273 (12) Å for S12]. Moreover, the outer ethyl­ene groups tend to be more eclipsed in the mol­ecule of the dimer whereas they tend to be more staggered in the other mol­ecule (Fig. 3 ▸). All these features are similar to those reported for the (BEDT-TTF)_4_[*AM*(C_2_O_4_)_3_]·solv. family.

Contrary to what is observed in other BEDT-TTF compounds associated with [Cu(NCS)_*x*_] anions that are present as polymeric [Cu(NCS)_*x*_] entities, in the title compound discrete [Cu(NCS)_4_]^2–^ units are observed. The Cu^II^ cation (which lies on a centre of inversion) of the [Cu(NCS)_4_]^2–^ dianion adopts an almost regular square-planar CuN_4_ environment, with N—Cu—N angles close to 90 and 180°. Intra­molecular bond lengths in the anion are in agreement with other tetra­thio­cyanato­cuprates(II) (Wang *et al.*, 2008 ▸; Chekhlov, 2009 ▸). It should be noted that one Cu—NCS fragment is more bent than the other one [angle Cu—N2—C2 of 151.6 (4)° *versus* 175.2 (3)° for Cu—N1—C1; Fig. 2 ▸].

## Supra­molecular features   

**Figure 4:**
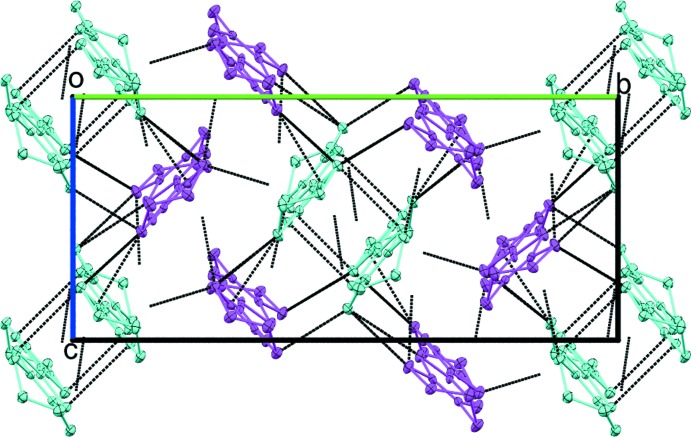
View along [100] of the packing of the mol­ecular entities in the crystal structure of (BEDT-TTF)_4_[Cu(NCS)_4_]. The dimer of BEDT-TTF mol­ecules (turquoise) is surrounded by other BEDT-TTF mol­ecules (purple). Black dotted lines represent short S⋯S and S⋯H contacts shorter than the sum of the van der Waals radii (for numerical details, see: Table 1 ▸).

**Figure 5:**
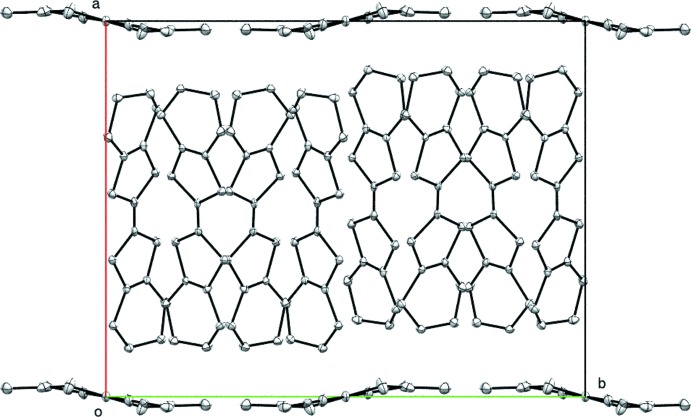
View of the structural arrangement of (BEDT-TTF)_4_[Cu(NCS)_4_], showing layers of BEDT-TTF mol­ecules parallel to (001) separated by layers of [Cu(NCS)_4_]^2–^ dianions.

The BEDT-TTF mol­ecules in the dimer stack face-to-face. The inter­planar distance within the dimer is 3.62 (3) Å, considering the least-squares planes of the mol­ecule except for the terminal ethyl­ene groups. In addition, there are two pairs of short S⋯S contacts within the dimer [S5⋯S8 = 3.461 (1) and S6⋯S7 = 3.515 (1) Å]. Each dimer is surrounded by six adjacent BEDT-TTF mol­ecules (Fig. 4 ▸). Four of them lie nearly perpendicular to the dimer [angle of 86.16 (2)°] and are connected with the dimer through short S⋯S contacts (Table 1 ▸) and the two others are almost parallel to the dimer [angle of 7.09 (4)°] with short S⋯H contacts (Table 1 ▸). This arrangement leads to layers of BEDT-TTF donors, extending parallel to (001). The layers have a width that corresponds to the length of the *a* axis and are separated from each other by layers of [Cu(NCS)_4_]^2–^ dianions (Fig. 5 ▸).

## Synthesis and crystallization   

(BEDT-TTF)_4_[Cu(NCS)_4_] was prepared by galvanostatic electrocrystallization in an H-shaped cell, equipped with Pt electrodes and with a glass frit between the anodic and cathodic compartments. To the cathodic compartment were added EMIM(SCN) (EMIM = 1-ethyl-3-methyl­imidazolium; 60 µl, 0.4 mmol) and freshly distilled 1,1,2-TCE (TCE = trichlorethyl­ene; 10 ml). Cu(NCS) (8 mg, 0.07 mmol) and EMIM(SCN) (60 µL, 0.4 mmoL) were added to the anodic compartment, which was immediately filled with BEDT-TTF (30 mg, 0.08 mmol), previously dissolved in 10 ml of 1,1,2-TCE at 343 K. The current was set at 100 µA (current density of 318 µA cm^−2^). After 12 h, a black powder corresponding to the desired compound (BEDT-TTF)_2_[Cu(NCS)_2_] was harvested by filtration, together with some crystals of (BEDT-TTF)_4_[Cu(NCS)_4_], which were washed with 1,1,2-TCE and dried under vacuum.

## Refinement   

Crystal data, data collection and structure refinement details are summarized in Table 2 ▸. The crystal diffracted rather weakly (2*θ*_max_ = 47.42°). The hydrogen atoms of the ethyl­ene bridges were placed in idealized positions and were refined with C—H = 0.99 Å and with *U*_iso_(H) = 1.2*U*_eq_(C). Reflections (100) and (110) were obstructed by the beam stop and thus were excluded from the refinement.

## Supplementary Material

Crystal structure: contains datablock(s) I. DOI: 10.1107/S2056989018015293/wm5468sup1.cif

Structure factors: contains datablock(s) I. DOI: 10.1107/S2056989018015293/wm5468Isup2.hkl

CCDC reference: 1876009

Additional supporting information:  crystallographic information; 3D view; checkCIF report

## Figures and Tables

**Table 1:** Table of contacts (Å) shorter than the sum of the van der Waals radii

Atom 1⋯atom 2	Length	Symmetry operation on atom 2	
S5⋯S8	3.461 (1)	1 − *x*, 1 − *y*, 1 − *z*	
S6⋯S7	3.515 (1)	1 − *x*, 1 − *y*, 1 − *z*	
S4⋯S12	3.463 (1)	*x*, *y*, *z*	
S4⋯S14	3.586 (1)	*x*, *y*, *z*	
S10⋯H21*B*	2.76	*x*, *y*, *z*	
H12*A*⋯S17	2.81	*x*, *y*, 1 + *z*	
S3⋯S11	3.511 (1)	*x*, *y*, 1 + *z*	
S5⋯S11	3.508 (1)	*x*, *y*, 1 + *z*	
S9⋯S15	3.571 (1)	*x*, *y*, 1 + *z*	
S9⋯S17	3.574 (1)	*x*, *y*, 1 + *z*	
S3⋯S17	3.470 (1)	1 − *x*, 1 − *y*, 1 − *z*	
S5⋯S17	3.533 (1)	1 − *x*, 1 − *y*, 1 − *z*	
S9⋯S11	3.522 (1)	1 − *x*, 1 − *y*, 1 − *z*	
H4*B*⋯S12	2.74	*x*, 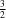 − *y*, 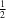 + *z*	
S12⋯H14*A*	2.73	*x*, 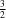 − *y*, 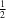 + *z*	

**Table 2:** Experimental details

Crystal data	
Chemical formula	(C_10_H_8_S_8_)_2_[Cu(*CNS*)_4_]·2C_10_H_8_S_8_
*M*_r_	1834.43
Crystal system, space group	Monoclinic, *P*2_1_/*c*
Temperature (K)	100
*a*, *b*, *c* (Å)	16.9036 (17), 21.004 (2), 9.6205 (9)
β (°)	103.071 (3)
*V* (Å^3^)	3327.1 (6)
*Z*	2
Radiation type	Mo *K*α
μ (mm^−1^)	1.50
Crystal size (mm)	0.18 × 0.16 × 0.02

Data collection	
Diffractometer	Bruker Kappa APEXII Quazar CCD
Absorption correction	Multi-scan (*SADABS*; Bruker, 2012 ▸)
*T*_min_, *T*_max_	0.652, 0.745
No. of measured, independent and observed [*I* > 2σ(*I*)] reflections	48335, 5028, 3816
*R*_int_	0.070
θ_max_ (°)	23.7
(sin θ/λ)_max_ (Å^−1^)	0.566

Refinement	
*R*[*F* ^2^ > 2σ(*F* ^2^)], *wR*(*F* ^2^), *S*	0.032, 0.078, 1.02
No. of reflections	5028
No. of parameters	385
H-atom treatment	H-atom parameters constrained
Δρ_max_, Δρ_min_ (e Å^−3^)	0.38, −0.39

Computer programs: *APEX2* and *SAINT* (Bruker, 2012 ▸), *SHELXS97* (Sheldrick, 2008 ▸), *SHELXL2018* (Sheldrick, 2015 ▸), *Mercury* (Macrae *et al.*, 2006 ▸) and *WinGX* (Farrugia, 2012 ▸).

## supplementary crystallographic information

### Crystal data

**Table:**

(C_10_H_8_S_8_)_2_[Cu(CNS)_4_]·2C_10_H_8_S_8_	*F*(000) = 1858
*M**_r_* = 1834.43	*D*_x_ = 1.831 Mg m^−^^3^
Monoclinic, *P*2_1_/*c*	Mo *K*α radiation, λ = 0.71073 Å
*a* = 16.9036 (17) Å	Cell parameters from 7249 reflections
*b* = 21.004 (2) Å	θ = 2.4–23.5°
*c* = 9.6205 (9) Å	µ = 1.50 mm^−^^1^
β = 103.071 (3)°	*T* = 100 K
*V* = 3327.1 (6) Å^3^	Plate, orange
*Z* = 2	0.18 × 0.16 × 0.02 mm

### Data collection

**Table:**

Bruker Kappa APEXII Quazar CCD diffractometer	5028 independent reflections
Radiation source: microfocus sealed tube	3816 reflections with *I* > 2σ(*I*)
Multilayer optics monochromator	*R*_int_ = 0.070
phi and ω scans	θ_max_ = 23.7°, θ_min_ = 2.4°
Absorption correction: multi-scan (*SADABS*; Bruker, 2012)	*h* = −19→19
*T*_min_ = 0.652, *T*_max_ = 0.745	*k* = −23→23
48335 measured reflections	*l* = −10→10

### Refinement

**Table:**

Refinement on *F*^2^	0 restraints
Least-squares matrix: full	Hydrogen site location: inferred from neighbouring sites
*R*[*F*^2^ > 2σ(*F*^2^)] = 0.032	H-atom parameters constrained
*wR*(*F*^2^) = 0.078	*w* = 1/[σ^2^(*F*_o_^2^) + (0.0295*P*)^2^ + 4.1709*P*] where *P* = (*F*_o_^2^ + 2*F*_c_^2^)/3
*S* = 1.02	(Δ/σ)_max_ = 0.001
5028 reflections	Δρ_max_ = 0.38 e Å^−^^3^
385 parameters	Δρ_min_ = −0.39 e Å^−^^3^

### Special details

**Table:**

Geometry. All esds (except the esd in the dihedral angle between two l.s. planes) are estimated using the full covariance matrix. The cell esds are taken into account individually in the estimation of esds in distances, angles and torsion angles; correlations between esds in cell parameters are only used when they are defined by crystal symmetry. An approximate (isotropic) treatment of cell esds is used for estimating esds involving l.s. planes.

### Fractional atomic coordinates and isotropic or equivalent isotropic displacement parameters (Å^2^)

**Table:**

	*x*	*y*	*z*	*U*_iso_*/*U*_eq_	
C1	−0.0171 (2)	0.42002 (18)	0.7252 (4)	0.0213 (9)	
C2	0.0246 (2)	0.6302 (2)	0.8878 (4)	0.0253 (10)	
C3	0.8700 (2)	0.54753 (16)	0.5992 (4)	0.0187 (9)	
H3A	0.928155	0.545997	0.647921	0.022*	
H3B	0.862454	0.520423	0.512981	0.022*	
C4	0.8478 (2)	0.61543 (17)	0.5535 (4)	0.0209 (9)	
H4A	0.890091	0.632876	0.507824	0.025*	
H4B	0.847851	0.641325	0.639476	0.025*	
C5	0.7131 (2)	0.54291 (16)	0.6393 (4)	0.0149 (8)	
C6	0.6896 (2)	0.58496 (16)	0.5308 (4)	0.0160 (8)	
C7	0.5595 (2)	0.55932 (15)	0.6157 (4)	0.0149 (8)	
C8	0.4827 (2)	0.55887 (16)	0.6416 (4)	0.0154 (8)	
C9	0.3595 (2)	0.53724 (16)	0.7503 (4)	0.0154 (8)	
C10	0.3323 (2)	0.57822 (16)	0.6408 (4)	0.0160 (8)	
C11	0.2046 (2)	0.52444 (17)	0.7908 (4)	0.0209 (9)	
H11A	0.184487	0.494450	0.711275	0.025*	
H11B	0.171329	0.518267	0.862179	0.025*	
C12	0.1924 (2)	0.59208 (17)	0.7337 (4)	0.0209 (9)	
H12A	0.217762	0.621861	0.810709	0.025*	
H12B	0.133437	0.601329	0.708352	0.025*	
C13	0.8509 (2)	0.64170 (17)	0.0556 (4)	0.0215 (9)	
H13A	0.858052	0.616445	0.144492	0.026*	
H13B	0.892041	0.626958	0.004150	0.026*	
C14	0.8670 (2)	0.71118 (17)	0.0950 (4)	0.0203 (9)	
H14A	0.852389	0.737338	0.007359	0.024*	
H14B	0.925749	0.716993	0.136028	0.024*	
C15	0.6912 (2)	0.66642 (16)	0.0439 (4)	0.0132 (8)	
C16	0.7133 (2)	0.71147 (16)	0.1441 (4)	0.0156 (8)	
C17	0.5605 (2)	0.69326 (16)	0.1286 (4)	0.0169 (9)	
C18	0.4857 (2)	0.69108 (16)	0.1551 (4)	0.0165 (9)	
C19	0.3385 (2)	0.65929 (16)	0.1588 (4)	0.0141 (8)	
C20	0.3569 (2)	0.70712 (16)	0.2528 (4)	0.0149 (8)	
C21	0.1796 (2)	0.65689 (16)	0.2003 (4)	0.0168 (9)	
H21A	0.123433	0.652063	0.142740	0.020*	
H21B	0.182206	0.635883	0.293364	0.020*	
C22	0.1970 (2)	0.72687 (17)	0.2261 (4)	0.0229 (9)	
H22A	0.152515	0.746340	0.263158	0.028*	
H22B	0.197940	0.747725	0.134226	0.028*	
S1	−0.02733 (6)	0.37420 (5)	0.58614 (11)	0.0285 (3)	
S2	0.02204 (7)	0.70695 (5)	0.86453 (12)	0.0331 (3)	
S3	0.81075 (6)	0.51479 (4)	0.71701 (10)	0.0169 (2)	
S4	0.75024 (6)	0.62342 (4)	0.43121 (10)	0.0210 (2)	
S5	0.63665 (5)	0.51340 (4)	0.71623 (10)	0.0158 (2)	
S6	0.58730 (6)	0.60578 (4)	0.48575 (10)	0.0173 (2)	
S7	0.46026 (6)	0.51311 (4)	0.77698 (10)	0.0178 (2)	
S8	0.40302 (6)	0.60313 (4)	0.54464 (10)	0.0167 (2)	
S9	0.30860 (6)	0.50474 (4)	0.87210 (10)	0.0198 (2)	
S10	0.23390 (6)	0.60719 (4)	0.57979 (10)	0.0198 (2)	
S11	0.75135 (6)	0.62589 (4)	−0.05352 (10)	0.0183 (2)	
S12	0.81090 (6)	0.73927 (4)	0.22123 (10)	0.0178 (2)	
S13	0.58828 (6)	0.64487 (4)	−0.00163 (10)	0.0180 (2)	
S14	0.63616 (6)	0.74608 (4)	0.21522 (10)	0.0183 (2)	
S15	0.41224 (6)	0.63676 (4)	0.06567 (10)	0.0180 (2)	
S16	0.45292 (6)	0.74247 (4)	0.27426 (11)	0.0212 (2)	
S17	0.24819 (6)	0.61583 (4)	0.10988 (11)	0.0208 (2)	
S18	0.29242 (6)	0.74148 (5)	0.35111 (10)	0.0232 (2)	
Cu1	0.000000	0.500000	1.000000	0.02233 (18)	
N1	−0.0100 (2)	0.45238 (15)	0.8251 (4)	0.0282 (8)	
N2	0.0279 (2)	0.57616 (18)	0.9071 (4)	0.0379 (10)	

### Atomic displacement parameters (Å^2^)

**Table:**

	*U*^11^	*U*^22^	*U*^33^	*U*^12^	*U*^13^	*U*^23^
C1	0.015 (2)	0.017 (2)	0.030 (2)	0.0038 (17)	0.0001 (19)	0.0093 (19)
C2	0.019 (2)	0.037 (3)	0.021 (2)	−0.0072 (19)	0.0067 (19)	−0.005 (2)
C3	0.019 (2)	0.017 (2)	0.021 (2)	0.0019 (16)	0.0063 (18)	0.0027 (16)
C4	0.016 (2)	0.016 (2)	0.030 (2)	−0.0010 (16)	0.0057 (18)	0.0024 (17)
C5	0.019 (2)	0.0108 (19)	0.016 (2)	0.0001 (16)	0.0054 (17)	−0.0015 (16)
C6	0.018 (2)	0.0103 (19)	0.020 (2)	−0.0008 (16)	0.0063 (17)	−0.0007 (16)
C7	0.020 (2)	0.0085 (18)	0.014 (2)	0.0001 (16)	0.0009 (17)	−0.0022 (15)
C8	0.021 (2)	0.0120 (19)	0.0123 (19)	0.0009 (16)	0.0007 (17)	0.0001 (15)
C9	0.018 (2)	0.0137 (19)	0.015 (2)	−0.0001 (16)	0.0038 (17)	−0.0028 (16)
C10	0.022 (2)	0.0123 (19)	0.014 (2)	0.0002 (16)	0.0058 (17)	−0.0027 (16)
C11	0.018 (2)	0.024 (2)	0.022 (2)	−0.0023 (17)	0.0077 (18)	0.0015 (17)
C12	0.022 (2)	0.019 (2)	0.023 (2)	0.0047 (17)	0.0083 (18)	0.0018 (17)
C13	0.016 (2)	0.023 (2)	0.026 (2)	0.0008 (17)	0.0075 (18)	−0.0040 (18)
C14	0.017 (2)	0.024 (2)	0.021 (2)	−0.0037 (17)	0.0063 (18)	−0.0055 (17)
C15	0.014 (2)	0.0102 (19)	0.017 (2)	0.0009 (15)	0.0056 (16)	0.0041 (16)
C16	0.016 (2)	0.016 (2)	0.016 (2)	−0.0024 (16)	0.0046 (17)	0.0025 (16)
C17	0.017 (2)	0.0100 (19)	0.021 (2)	0.0014 (16)	−0.0003 (18)	−0.0036 (16)
C18	0.018 (2)	0.0079 (19)	0.022 (2)	−0.0009 (16)	0.0029 (18)	−0.0031 (16)
C19	0.014 (2)	0.0114 (19)	0.016 (2)	0.0010 (15)	0.0025 (17)	0.0051 (16)
C20	0.018 (2)	0.0107 (19)	0.015 (2)	−0.0001 (15)	0.0014 (17)	0.0026 (15)
C21	0.013 (2)	0.020 (2)	0.019 (2)	−0.0009 (16)	0.0042 (17)	−0.0043 (16)
C22	0.019 (2)	0.019 (2)	0.031 (2)	−0.0010 (17)	0.0078 (19)	−0.0043 (18)
S1	0.0236 (6)	0.0350 (6)	0.0274 (6)	0.0033 (5)	0.0066 (5)	−0.0062 (5)
S2	0.0253 (6)	0.0306 (6)	0.0430 (7)	0.0026 (5)	0.0070 (5)	0.0175 (5)
S3	0.0160 (5)	0.0153 (5)	0.0196 (5)	0.0019 (4)	0.0045 (4)	0.0046 (4)
S4	0.0236 (6)	0.0184 (5)	0.0222 (6)	0.0021 (4)	0.0079 (5)	0.0087 (4)
S5	0.0155 (5)	0.0143 (5)	0.0170 (5)	0.0004 (4)	0.0027 (4)	0.0033 (4)
S6	0.0195 (6)	0.0141 (5)	0.0183 (5)	0.0024 (4)	0.0041 (4)	0.0033 (4)
S7	0.0188 (5)	0.0169 (5)	0.0175 (5)	0.0019 (4)	0.0037 (4)	0.0032 (4)
S8	0.0173 (5)	0.0146 (5)	0.0180 (5)	0.0018 (4)	0.0034 (4)	0.0031 (4)
S9	0.0217 (6)	0.0186 (5)	0.0199 (5)	0.0014 (4)	0.0065 (4)	0.0051 (4)
S10	0.0189 (6)	0.0222 (5)	0.0185 (5)	0.0052 (4)	0.0046 (4)	0.0044 (4)
S11	0.0172 (5)	0.0180 (5)	0.0209 (5)	−0.0026 (4)	0.0067 (4)	−0.0048 (4)
S12	0.0160 (5)	0.0194 (5)	0.0180 (5)	−0.0045 (4)	0.0041 (4)	−0.0033 (4)
S13	0.0139 (5)	0.0148 (5)	0.0243 (6)	−0.0003 (4)	0.0024 (4)	−0.0042 (4)
S14	0.0151 (5)	0.0135 (5)	0.0259 (5)	−0.0006 (4)	0.0038 (4)	−0.0049 (4)
S15	0.0142 (5)	0.0156 (5)	0.0242 (5)	−0.0017 (4)	0.0045 (4)	−0.0062 (4)
S16	0.0163 (5)	0.0179 (5)	0.0300 (6)	−0.0027 (4)	0.0066 (5)	−0.0093 (4)
S17	0.0200 (6)	0.0175 (5)	0.0272 (6)	−0.0061 (4)	0.0099 (5)	−0.0074 (4)
S18	0.0221 (6)	0.0239 (5)	0.0260 (6)	−0.0060 (4)	0.0102 (5)	−0.0116 (4)
Cu1	0.0214 (4)	0.0135 (3)	0.0321 (4)	−0.0006 (3)	0.0061 (3)	−0.0034 (3)
N1	0.033 (2)	0.0178 (18)	0.032 (2)	0.0032 (16)	0.0023 (17)	0.0016 (17)
N2	0.053 (3)	0.026 (2)	0.041 (2)	−0.0139 (19)	0.024 (2)	−0.0099 (18)

### Geometric parameters (Å, º)

**Table:**

C1—N1	1.161 (5)	C14—H14A	0.9900
C1—S1	1.625 (4)	C14—H14B	0.9900
C2—N2	1.149 (5)	C15—C16	1.342 (5)
C2—S2	1.627 (5)	C15—S11	1.752 (4)
C3—C4	1.514 (5)	C15—S13	1.755 (4)
C3—S3	1.809 (4)	C16—S12	1.749 (4)
C3—H3A	0.9900	C16—S14	1.761 (4)
C3—H3B	0.9900	C17—C18	1.346 (5)
C4—S4	1.804 (4)	C17—S14	1.755 (4)
C4—H4A	0.9900	C17—S13	1.758 (4)
C4—H4B	0.9900	C18—S16	1.753 (4)
C5—C6	1.357 (5)	C18—S15	1.761 (4)
C5—S5	1.742 (4)	C19—C20	1.340 (5)
C5—S3	1.753 (4)	C19—S17	1.749 (4)
C6—S6	1.740 (4)	C19—S15	1.757 (4)
C6—S4	1.751 (4)	C20—S18	1.752 (4)
C7—C8	1.377 (5)	C20—S16	1.755 (4)
C7—S5	1.731 (4)	C21—C22	1.508 (5)
C7—S6	1.732 (4)	C21—S17	1.817 (4)
C8—S8	1.726 (4)	C21—H21A	0.9900
C8—S7	1.728 (4)	C21—H21B	0.9900
C9—C10	1.358 (5)	C22—S18	1.807 (4)
C9—S7	1.740 (4)	C22—H22A	0.9900
C9—S9	1.742 (4)	C22—H22B	0.9900
C10—S10	1.743 (4)	S3—S17^i^	3.4702 (13)
C10—S8	1.749 (4)	S3—S11^ii^	3.5113 (13)
C11—C12	1.520 (5)	S4—S12	3.4627 (13)
C11—S9	1.803 (4)	S4—S14	3.5862 (13)
C11—H11A	0.9900	S5—S11^ii^	3.5079 (13)
C11—H11B	0.9900	S5—S17^i^	3.5330 (13)
C12—S10	1.805 (4)	S6—S7^i^	3.5146 (13)
C12—H12A	0.9900	S9—S11^i^	3.5216 (13)
C12—H12B	0.9900	S9—S15^ii^	3.5707 (13)
C13—C14	1.517 (5)	S9—S17^ii^	3.5743 (14)
C13—S11	1.802 (4)	Cu1—N1	1.932 (4)
C13—H13A	0.9900	Cu1—N1^iii^	1.932 (4)
C13—H13B	0.9900	Cu1—N2^iii^	1.942 (4)
C14—S12	1.800 (4)	Cu1—N2	1.942 (4)
			
N1—C1—S1	179.5 (4)	C19—C20—S18	126.5 (3)
N2—C2—S2	178.3 (4)	C19—C20—S16	117.6 (3)
C4—C3—S3	113.8 (3)	S18—C20—S16	115.7 (2)
C4—C3—H3A	108.8	C22—C21—S17	114.8 (3)
S3—C3—H3A	108.8	C22—C21—H21A	108.6
C4—C3—H3B	108.8	S17—C21—H21A	108.6
S3—C3—H3B	108.8	C22—C21—H21B	108.6
H3A—C3—H3B	107.7	S17—C21—H21B	108.6
C3—C4—S4	114.0 (3)	H21A—C21—H21B	107.5
C3—C4—H4A	108.8	C21—C22—S18	112.7 (3)
S4—C4—H4A	108.8	C21—C22—H22A	109.0
C3—C4—H4B	108.8	S18—C22—H22A	109.0
S4—C4—H4B	108.8	C21—C22—H22B	109.0
H4A—C4—H4B	107.7	S18—C22—H22B	109.0
C6—C5—S5	116.2 (3)	H22A—C22—H22B	107.8
C6—C5—S3	129.2 (3)	C5—S3—C3	101.87 (17)
S5—C5—S3	114.62 (19)	C5—S3—S17^i^	97.25 (12)
C5—C6—S6	117.2 (3)	C3—S3—S17^i^	149.40 (12)
C5—C6—S4	128.0 (3)	C5—S3—S11^ii^	70.65 (12)
S6—C6—S4	114.8 (2)	C3—S3—S11^ii^	114.76 (12)
C8—C7—S5	121.2 (3)	S17^i^—S3—S11^ii^	94.00 (3)
C8—C7—S6	123.7 (3)	C6—S4—C4	99.35 (18)
S5—C7—S6	115.1 (2)	C6—S4—S12	158.54 (12)
C7—C8—S8	123.5 (3)	C4—S4—S12	95.63 (13)
C7—C8—S7	121.1 (3)	C6—S4—S14	110.11 (12)
S8—C8—S7	115.4 (2)	C4—S4—S14	137.49 (12)
C10—C9—S7	116.6 (3)	S12—S4—S14	49.34 (3)
C10—C9—S9	129.6 (3)	C7—S5—C5	95.85 (17)
S7—C9—S9	113.8 (2)	C7—S5—S11^ii^	102.50 (12)
C9—C10—S10	127.8 (3)	C5—S5—S11^ii^	70.81 (12)
C9—C10—S8	116.6 (3)	C7—S5—S17^i^	163.25 (12)
S10—C10—S8	115.6 (2)	C5—S5—S17^i^	95.28 (12)
C12—C11—S9	114.3 (3)	S11^ii^—S5—S17^i^	92.97 (3)
C12—C11—H11A	108.7	C7—S6—C6	95.43 (17)
S9—C11—H11A	108.7	C7—S6—S7^i^	93.63 (12)
C12—C11—H11B	108.7	C6—S6—S7^i^	93.06 (12)
S9—C11—H11B	108.7	C8—S7—C9	95.80 (17)
H11A—C11—H11B	107.6	C8—S8—C10	95.53 (17)
C11—C12—S10	114.5 (3)	C8—S8—S5^i^	90.59 (12)
C11—C12—H12A	108.6	C10—S8—S5^i^	97.38 (12)
S10—C12—H12A	108.6	C9—S9—C11	101.52 (17)
C11—C12—H12B	108.6	C9—S9—S11^i^	151.62 (12)
S10—C12—H12B	108.6	C11—S9—S11^i^	91.85 (12)
H12A—C12—H12B	107.6	C9—S9—S15^ii^	77.75 (12)
C14—C13—S11	114.4 (3)	C11—S9—S15^ii^	111.30 (13)
C14—C13—H13A	108.6	S11^i^—S9—S15^ii^	120.58 (3)
S11—C13—H13A	108.6	C9—S9—S17^ii^	115.51 (12)
C14—C13—H13B	108.6	C11—S9—S17^ii^	74.71 (13)
S11—C13—H13B	108.6	S11^i^—S9—S17^ii^	92.03 (3)
H13A—C13—H13B	107.6	S15^ii^—S9—S17^ii^	48.46 (3)
C13—C14—S12	113.1 (3)	C10—S10—C12	100.41 (17)
C13—C14—H14A	109.0	C15—S11—C13	100.20 (17)
S12—C14—H14A	109.0	C16—S12—C14	101.22 (17)
C13—C14—H14B	109.0	C16—S12—S4	68.74 (12)
S12—C14—H14B	109.0	C14—S12—S4	115.70 (13)
H14A—C14—H14B	107.8	C15—S13—C17	94.73 (17)
C16—C15—S11	128.8 (3)	C17—S14—C16	94.53 (17)
C16—C15—S13	117.4 (3)	C17—S14—S4	93.43 (12)
S11—C15—S13	113.79 (19)	C16—S14—S4	65.17 (11)
C15—C16—S12	128.5 (3)	C19—S15—C18	94.55 (17)
C15—C16—S14	117.4 (3)	C18—S16—C20	94.76 (17)
S12—C16—S14	114.1 (2)	C19—S17—C21	103.65 (17)
C18—C17—S14	123.1 (3)	C20—S18—C22	98.12 (17)
C18—C17—S13	122.0 (3)	N1—Cu1—N1^iii^	180.0
S14—C17—S13	114.9 (2)	N1—Cu1—N2^iii^	89.53 (14)
C17—C18—S16	123.5 (3)	N1^iii^—Cu1—N2^iii^	90.47 (14)
C17—C18—S15	121.2 (3)	N1—Cu1—N2	90.46 (14)
S16—C18—S15	115.2 (2)	N1^iii^—Cu1—N2	89.53 (14)
C20—C19—S17	128.9 (3)	N2^iii^—Cu1—N2	180.0
C20—C19—S15	117.6 (3)	C1—N1—Cu1	175.2 (3)
S17—C19—S15	113.5 (2)	C2—N2—Cu1	151.6 (4)

Symmetry codes: (i) −*x*+1, −*y*+1, −*z*+1; (ii) *x*, *y*, *z*+1; (iii) −*x*, −*y*+1, −*z*+2.
